# QEKI: A Quantum–Classical Framework for Efficient Bayesian Inversion of PDEs

**DOI:** 10.3390/e28020156

**Published:** 2026-01-30

**Authors:** Jiawei Yong, Sihai Tang

**Affiliations:** School of Information Science and Technology, ShanghaiTech University, Shanghai 201210, China; yongjw2023@shanghaitech.edu.cn

**Keywords:** physics-informed neural networks, quantum neural networks, ensemble Kalman inversion, noisy data

## Abstract

Solving Bayesian inverse problems efficiently stands as a major bottleneck in scientific computing. Although Bayesian Physics-Informed Neural Networks (B-PINNs) have introduced a robust way to quantify uncertainty, the high-dimensional parameter spaces inherent in deep learning often lead to prohibitive sampling costs. Addressing this, our work introduces Quantum-Encodable Bayesian PINNs trained via Classical Ensemble Kalman Inversion (QEKI), a framework that pairs Quantum Neural Networks (QNNs) with Ensemble Kalman Inversion (EKI). The core advantage lies in the QNN’s ability to act as a compact surrogate for PDE solutions, capturing complex physics with significantly fewer parameters than classical networks. By adopting the gradient-free EKI for training, we mitigate the barren plateau issue that plagues quantum optimization. Through several benchmarks on 1D and 2D nonlinear PDEs, we show that QEKI yields precise inversions and substantial parameter compression, even in the presence of noise. While large-scale applications are constrained by current quantum hardware, this research outlines a viable hybrid framework for including quantum features within Bayesian uncertainty quantification.

## 1. Introduction

Partial Differential Equations (PDEs) are important tools for describing the dynamics of physical, chemical, and engineering systems. In addition to state variables, these models often depend on multiple parameters that characterize material properties, source terms, or boundary conditions. In many real-world scenarios, some of these parameters are often unknown and must be inferred from limited and noisy measurement data, which are often referred to as PDE inverse problems.

Physics-Informed Neural Networks (PINNs) [1,2] provide a flexible framework for solving partial differential equations by embedding the governing physical laws into the loss function. This formulation has been widely used in both forward and inverse PDE problems. In parallel, operator learning methods have been developed to approximate mappings between function spaces. DeepONet [3] adopts a dual-network architecture, in which a branch network encodes input functions and a trunk network represents spatial coordinates, enabling the approximation of nonlinear operators. Another representative approach is the Fourier Neural Operator (FNO) [4], which parameterizes integral kernels in the frequency domain using Fourier representations and has been applied to a range of problems involving complex physical systems.

To further enable uncertainty quantification and Bayesian inference, the Bayesian Physics-Informed Neural Network (B-PINNs) framework [5] extends PINNs by treating network parameters probabilistically. B-PINNs originally rely on Hamiltonian Monte Carlo (HMC), which provides asymptotically exact posterior samples but is often computationally expensive. More recent work [6,7] instead adopts the Ensemble Kalman Inversion (EKI) [8,9], a gradient-free and efficient alternative that approximates posterior updates through ensemble-based dynamics.

The rise of Quantum Neural Networks (QNNs) [10,11] has brought new opportunities for deep learning and scientific computing. QNNs achieve a nonlinear representation and probabilistic modeling in a high-dimensional Hilbert space through a quantum feature mapping and variational quantum circuits. The strength of QNNs comes from their ability to combine quantum superposition with the intrinsic parallelism of neural networks, enabling a computational framework with significant potential [12,13]. It has been demonstrated in Ref. [14] that quantum models capable of realizing all sets of Fourier coefficients can act as simulators of universal functions. Several recent studies [15,16,17] have begun to explore integrating QNNs with Physics-Informed Neural Networks. However, training QNNs with gradient-based methods can suffer from the barren plateau phenomenon [18,19], where gradients vanish exponentially with the number of qubits or the depth of the circuit, posing challenges for optimization. Although previous research [20,21,22] has yielded a range of effective algorithms to mitigate this phenomenon, the quest for ever-improving, highly effective solutions remains of paramount importance.

Motivated by these developments, this paper proposes a Quantum-Encodable Bayesian PINNs trained via Classical Ensemble Kalman Inversion (QEKI) framework, which integrates QNNs into the traditional B-PINNs structure. Notably, the inherent structure of QNNs reduces the number of trainable parameters, which improves the representational efficiency of the inverse problem, allowing the architecture to achieve accurate posterior inference with fewer parameters. Furthermore, the EKI-based parameter update does not require gradient computation, which not only accelerates QNN training but also helps mitigate the barren plateau phenomenon. Although current experiments rely on simulated quantum devices, the accuracy and efficiency of the method are expected to improve further with the deployment of future quantum hardware.

The main contributions of this paper are summarized as follows:

(1) We propose a hybrid Quantum-Encodable Bayesian PINNs trained via Classical Ensemble Kalman Inversion (QEKI) framework that combines QNN-based agent modeling with EKI to achieve gradient-free training.
(2) We reduce the trainable parameters by more than an order of magnitude while achieving comparable accuracy. In addition, the method effectively avoids barren plateau behavior, offering a practical optimization method for QNNs.
(3) We provide numerical evidence on nonlinear Poisson, diffusion–reaction, and Burgers equations showing superior robustness to observation noise.

The remainder of the paper is organized as follows. In Section 2, we introduce the problem formulation, including the B-PINNs, HMC, EKI, and the principles of QNNs, and then present the algorithm of the proposed QEKI method. In Section 3, we describe the experimental setups and present the corresponding results for three representative test cases. Finally, in Section 4, we provide concluding remarks and discuss directions for future research.

## 2. Methodology

The problem we considered is formulated as follows.

(1) $$\begin{array}{cccc} N_{x}\left(u\left(x\right);\lambda \right) & =f(x), & \; & x\in D,\\ B_{x}\left(u\left(x\right);\lambda \right) & =b(x), & \; & x\in \partial D, \end{array}$$

where $N_{x}$ is the differential operator that describes the physical process, and $B_{x}$ is the boundary operator applied to the boundary. $D\subseteq R^{d}$ is the *d*-dimensional physical domain with boundary ∂D, and $\lambda \in R^{N_{\lambda }}$ represents a vector of unknown physical parameters. In addition, f(x) is the forcing function, b(x) is the boundary function, and u(x) is the solution of the PDE. The aim of the inverse problem is to infer the solution function u(x) and the unknown parameters λ by integrating observational data with physical equations. The available data consists of the following three sets:

(2) $$\begin{array}{c} D_{f}={\left\{x_{f}^{i},f^{i}\right\}}_{i=1}^{N_{f}},\;D_{b}={\left\{x_{b}^{i},b^{i}\right\}}_{i=1}^{N_{b}},\;D_{u}={\left\{x_{u}^{i},u^{i}\right\}}_{i=1}^{N_{u}}, \end{array}$$

where $x_{f},x_{u}\in D$ and $x_{b}\in \partial D$, $f_{i}$, $b_{i}$, $u_{i}$ correspond to residual data, boundary-condition data and solution measurement in points $x_{f}^{i},x_{b}^{i},x_{u}^{i}$, respectively, $N_{f}$, $N_{b}$, $N_{u}$ denote the number of residual, boundary and solution data points.

### 2.1. Bayesian Physics-Informed Neural Network

The Bayesian Physics-Informed Neural Network (B-PINNs) employs a fully connected neural network as a surrogate model $\tilde{u}\left(x;\theta \right)$ for the PDE solution u(x), where $\theta \in R^{N_{\theta }}$ represents the parameters of the neural network. Set $\xi =\left\{\theta ,\lambda \right\}$ collect both the surrogate model parameters and the unknown physical parameters. By Bayes’ theorem, the posterior distribution of ξ is conditioned on the solution measurements $D_{u}$, residual data $D_{f}$, and boundary data $D_{b}$, which can be obtained as follows:

(3) $$p\left(\xi |D_{u},D_{f},D_{b}\right)\propto p\left(\xi \right)p\left(D_{u}|\xi \right)p\left(D_{f}|\xi \right)p\left(D_{b}|\xi \right).$$

We assume that the parameters are independent and that θ follows a Gaussian distribution with zero mean. Therefore, we can express the prior as follows:

(4) $$p\left(\xi \right)=p\left(\lambda \right)p\left(\theta \right)=p\left(\lambda \right)\prod_{i=1}^{N_{\theta }}p\left(\theta^{i}\right),\;p\left(\theta^{i}\right)∼N\left(0,{\sigma_{\theta }^{i}}^{2}\right),$$

where $\sigma_{\theta }^{i}$ is the standard deviation of the corresponding neural network parameter $\theta_{i}$. In addition, the likelihood can be expressed as follows:

(5) $$\begin{array}{cc} p\left(D_{u}|\xi \right) & =\prod_{i=1}^{N_{u}}p\left(u^{i}|\xi \right),\;p\left(D_{f}|\xi \right)=\prod_{i=1}^{N_{f}}p\left(f^{i}|\xi \right),\;p\left(D_{b}|\xi \right)=\prod_{i=1}^{N_{b}}p\left(b^{i}|\xi \right),\\ p\left(u^{i}|\xi \right) & =\frac{1}{\sqrt{2\pi \sigma_{u}^{2}}}\exp\left(-\frac{{\left(u^{i}-\tilde{u}\left(x_{u}^{i};\theta \right)\right)}^{2}}{2\sigma_{u}^{2}}\right),\\ p\left(f^{i}|\xi \right) & =\frac{1}{\sqrt{2\pi \sigma_{f}^{2}}}\exp\left(-\frac{{\left(f^{i}-N_{x}\left(\tilde{u}\left(x_{f}^{i};\theta \right);\lambda \right)\right)}^{2}}{2\sigma_{f}^{2}}\right),\\ p\left(b^{i}|\xi \right) & =\frac{1}{\sqrt{2\pi \sigma_{b}^{2}}}\exp\left(-\frac{{\left(b^{i}-B_{x}\left(\tilde{u}\left(x_{b}^{i};\theta \right);\lambda \right)\right)}^{2}}{2\sigma_{b}^{2}}\right), \end{array}$$

where $\sigma_{u},\sigma_{f},\sigma_{b}$. The prior distribution of the physical parameters λ is problem-dependent. After obtaining the prior and likelihood functions, various sampling methods can be used to derive the posterior distribution. Within the B-PINNs framework, efficient posterior sampling is essential for parameter estimation. HMC enables thorough posterior exploration, while EKI provides a gradient-free iterative update. Excessive trainable parameters may reduce sampling accuracy and increase computational cost. QNNs can serve as a partial remedy by lowering the number of parameters, potentially improving sampling efficiency and estimation quality.

### 2.2. Hamiltonian Monte Carlo

Hamiltonian Monte Carlo (HMC) is an efficient Markov Chain Monte Carlo (MCMC) method specifically designed for sampling from complex high-dimensional probability distributions, and has been applied to inverse problems in Bayesian Neural Networks. By introducing auxiliary momentum variables and simulating Hamiltonian dynamics, HMC is capable of generating states that are widely separated while maintaining a high acceptance probability.

Suppose that the target posterior distribution of ξ conditioned on the observations $D_{u},D_{f},D_{b}$ is given by

(6) $$p\left(\xi |D_{u},D_{f},D_{b}\right)≃\exp\left(-V\left(\xi \right)\right),$$

where $V\left(\xi \right)=-\ln p\left(D_{u},D_{f},D_{b}|\xi \right)-\ln p\left(\xi \right)$ represents the potential energy. Then the Hamiltonian dynamics can be defined as follows:

(7) $$H\left(\xi ,r\right)=V\left(\xi \right)+\frac{1}{2}r^{T}M^{-1}r,$$

where r is an auxiliary momentum vector, and M is the corresponding mass matrix, which is set to be the identity matrix I. $\frac{1}{2}r^{T}M^{-1}r$ represents the kinetic energy. The Hamiltonian dynamics evolve the system according to

(8) $$\begin{array}{cc} d\xi & =-M^{-1}rdt,\\ dr & =-\nabla V(\xi )dt. \end{array}$$

We use the Leapfrog integration to perform updating, and the Metropolis–Hastings acceptance test determines whether the proposed sample is accepted. The complete HMC procedure is summarized in Algorithm 1.

### 2.3. Ensemble Kalman Inversion

Ensemble Kalman Inversion (EKI) is a gradient-free inversion method based on an ensemble of samples, originally developed from the Ensemble Kalman Filter (EnKF). Unlike traditional Bayesian inverse problem solutions, EKI does not require explicit gradient computation. Instead, it iteratively updates an ensemble of samples under observation constraints, progressively approximating the high-probability region of the posterior distribution.

For an inverse problem with known observation data $y_{\mathrm{obs}}$ and a forward model G, the goal is to estimate the unknown parameters ξ:

(9) $$y_{\mathrm{obs}}=G\left(\xi \right)+\eta ,$$

where η∼N(0,R) is the observation noise, and *R* is the observation covariance matrix. According to Bayes’ theorem, the posterior can be expressed in the following form:

(10) $$p\left(\xi |y_{\mathrm{obs}}\right)\propto p\left(y_{\mathrm{obs}}|\xi \right)p\left(\xi \right).$$

**Algorithm 1** Hamiltonian Monte Carlo (HMC)**Require:** initial states $\xi^{0}$, time step size δt, leapfrog steps *I*, total running steps *J*    **for** j=0,1,…,(J−1) **do**        Sample $r^{j}$ from N(0,M)        $\xi_{0}\leftarrow \xi^{j}$        $r_{0}\leftarrow r^{j}$        **for** i=0,1,…,(I−1) **do**            $r_{i}\leftarrow r_{i}-\frac{\delta t}{2}\nabla V\left(\xi_{i}\right)$            $\xi_{i+1}\leftarrow \xi_{i}+\delta tM^{-1}r_{i}$            $r_{i+1}\leftarrow r_{i}-\frac{\delta t}{2}\nabla V\left(\xi_{i+1}\right)$        **end for**        Sample p from U(0,1)        $\alpha \leftarrow \min\left[1,\exp\left(H\left(\xi_{I},r_{I}\right)-H\left(\xi^{j},r^{j}\right)\right)\right]$        **if** p≥α **then**            $\xi^{j+1}\leftarrow \xi_{I}$        **else**            $\xi^{j+1}\leftarrow \xi^{j}$        **end if**    **end for**    **Return:** $\xi^{1},\ldots ,\xi^{J}$

Assuming the prior distribution of the parameters as $\xi ∼N\left(\xi_{0},C_{0}\right)$, the likelihood can be expressed as

(11) $$p\left(y_{\mathrm{obs}}|\xi \right)\propto \exp\left(-\frac{{∥R^{-1/2}\left(y-G\left(\xi \right)\right)∥}_{2}^{2}}{2}\right).$$

To facilitate iterative inversion, we reformulate the Bayesian inverse problem as an artificial dynamical system:

(12) $$\begin{array}{cccc} \xi_{i} & =\xi_{i-1}+\epsilon_{i}, & \epsilon_{i} & ∼N(0,Q),\\ y_{i} & =G\left(\xi_{i}\right)+\eta_{i}, & \eta_{i} & ∼N(0,R). \end{array}$$

where $\epsilon_{i}$ represents an artificial parameter noise with covariance *Q* and $\eta_{i}$ denotes the observation error with covariance *R*. In this formulation, the parameters are treated as state variables that evolve incrementally through the iterative process, while the observation equations remain unchanged. To efficiently approximate the posterior distribution, EKI employs the Kalman gain formula for Gaussian posterior distributions to iteratively update the sample set as in Ref. [9]. Let ${\left\{\xi_{0}^{j}\right\}}_{j=1}^{J}$ denote the initial ensemble of *J* members drawn from the prior distribution. Then, at iteration *i*, the *j*-th ensemble member is updated as

(13) $$\xi_{i+1}^{j}=\xi_{i}^{j}+\epsilon_{i}^{j}+C_{i}^{\xi y}{\left(C_{i}^{yy}+R\right)}^{-1}\left(y_{\mathrm{obs}}+\eta_{i}^{j}-G\left(\xi_{i}^{j}\right)\right),$$

where $\epsilon_{i}^{j}$ and $\eta_{i}^{j}$ represent the corresponding parameter and the observation noise. The sample covariances are defined as

(14) $$\begin{array}{cc} C_{i}^{yy} & =\frac{1}{J-1}\sum_{j=1}^{J}\left(y_{i}^{j}-{\bar{y}}_{i}\right){\left(y_{i}^{j}-{\bar{y}}_{i}\right)}^{T},\\ C_{i}^{\xi y} & =\frac{1}{J-1}\sum_{j=1}^{J}\left(\xi_{i}^{j}-{\bar{\xi }}_{i}\right){\left(y_{i}^{j}-{\bar{y}}_{i}\right)}^{T}. \end{array}$$

At each iteration, the ensemble is updated according to the EKI scheme, producing a new set of parameter samples that progressively approximate the target posterior distribution.

### 2.4. Quantum Model

The quantum model $f_{\theta }\left(x\right)$ is formally defined as the expectation value of an observable *O* with respect to a state evolved by the quantum circuit [14]U(x,θ) as follows:

(15) $$f_{\theta }\left(x\right)=\langle 0|U^{†}\left(x,\theta \right)OU\left(x,\theta \right)|0\rangle ,$$

where |0〉 denotes the initial quantum state and U(x,θ) is the quantum circuit, which depends on the input x and the parameter θ, and takes the following form:

(16) $$U\left(x,\theta \right)=W^{\left(L+1\right)}\left(\theta \right)S\left(x\right)W^{\left(L\right)}\left(\theta \right)\ldots W^{\left(2\right)}\left(\theta \right)S\left(x\right)W^{\left(1\right)}\left(\theta \right).$$

**Figure 1:**
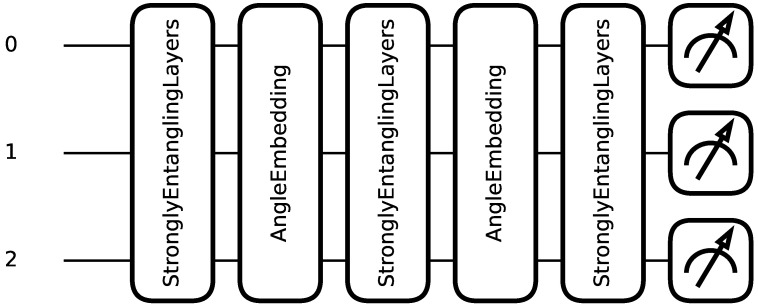
Quantum model architecture with 3 qubits, 2 sequential layers, and a measurement layer.

The quantum circuit is composed of sequential layers *L*, where each layer includes a data-encoding circuit block S(x) and a trainable variational circuit block W(θ). An example quantum circuit with 3 qubits and 2 sequential layers is shown in Figure 1.

The trainable and encoding layers of the quantum circuit are constructed using rotational gates [23]. The matrix representation of these gates is given below:

(17) $$\begin{array}{c} R_{X}\left(\theta \right)=\left(\begin{array}{cc} \cos\left(\frac{\theta }{2}\right) & -i\sin\left(\frac{\theta }{2}\right)\\ -i\sin\left(\frac{\theta }{2}\right) & \cos\left(\frac{\theta }{2}\right) \end{array}\right),\\ R_{Y}\left(\theta \right)=\left(\begin{array}{cc} \cos\left(\frac{\theta }{2}\right) & -\sin\left(\frac{\theta }{2}\right)\\ \sin\left(\frac{\theta }{2}\right) & \cos\left(\frac{\theta }{2}\right) \end{array}\right),\\ R_{Z}\left(\theta \right)=\left(\begin{array}{cc} \exp\left(-i\frac{\theta }{2}\right) & 0\\ 0 & \exp\left(i\frac{\theta }{2}\right) \end{array}\right). \end{array}$$

Applying these rotation gates to the basis states |0〉 and |1〉 produces:

(18) $$\begin{array}{cc} R_{X}\left(\theta \right)|0\rangle & =\cos\left(\frac{\theta }{2}\right)|0\rangle -i\sin\left(\frac{\theta }{2}\right)|1\rangle ,\\ R_{X}\left(\theta \right)|1\rangle & =-i\sin\left(\frac{\theta }{2}\right)|0\rangle +\cos\left(\frac{\theta }{2}\right)|1\rangle ,\\ R_{Y}\left(\theta \right)|0\rangle & =\cos\left(\frac{\theta }{2}\right)|0\rangle +\sin\left(\frac{\theta }{2}\right)|1\rangle ,\\ R_{Y}\left(\theta \right)|1\rangle & =-\sin\left(\frac{\theta }{2}\right)|0\rangle +\cos\left(\frac{\theta }{2}\right)|1\rangle ,\\ R_{Z}\left(\theta \right)|0\rangle & =\left[\cos\left(\frac{\theta }{2}\right)-i\sin\left(\frac{\theta }{2}\right)\right]|0\rangle ,\\ R_{Z}\left(\theta \right)|1\rangle & =\left[\cos\left(\frac{\theta }{2}\right)+i\sin\left(\frac{\theta }{2}\right)\right]|1\rangle . \end{array}$$

The data-encoding block S(x) is mathematically defined as a tensor product of rotations $R_{z}$:

(19) $$\begin{array}{c} S\left(x\right)=\overset{n}{\underset{i=1}{⨂}}R_{Z}\left(x_{i}\right) \end{array}$$

where *n* is the number of qubits and $x_{i}$ is the *i*-th component of the input vector x. Applying the encoding block to the initial *n*-qubit state ${⨂}_{i=1}^{n}|0\rangle$, we obtain the encoded state $|x\rangle ={⨂}_{i=1}^{n}|x_{i}\rangle$ as follows:

(20) $$\begin{array}{c} |x\rangle =S\left(x\right)\left(\overset{n}{\underset{i=1}{⨂}}|0\rangle \right)=\overset{n}{\underset{i=1}{⨂}}\left(R_{Z}\left(x_{i}\right)|0\rangle \right)=\overset{n}{\underset{i=1}{⨂}}\left(\left[\cos\left(\frac{x_{i}}{2}\right)-i\sin\left(\frac{x_{i}}{2}\right)\right]|0\rangle \right). \end{array}$$

**Figure 2:**
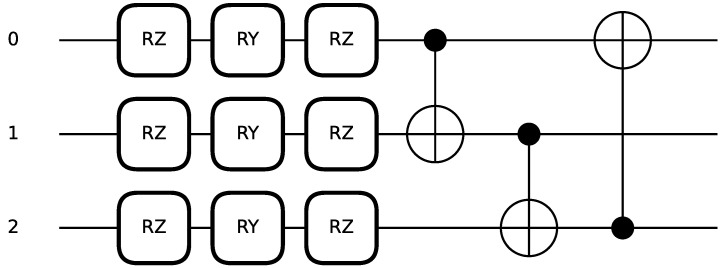
Circuit layout of a three-qubit strongly entangling layer using rotation gates and CNOT gates.

We use strongly entangling layers [24] as the trainable circuit block and training blocks W(θ) depend on the parameters θ that can be classically optimized. A strongly entangling layer with three qubits that contains 9 trainable variables takes the form as in Figure 2.

Applying the strongly entangling layer to the encoded state produces the following result:

(21) $$\begin{array}{c} W\left(\theta \right)|x\rangle =U_{CNOT}^{31}U_{CNOT}^{23}U_{CNOT}^{12}\left(\overset{3}{\underset{i=1}{⨂}}R_{Z}\left(\theta_{i}^{1}\right)R_{Y}\left(\theta_{i}^{2}\right)R_{Z}\left(\theta_{i}^{3}\right)|x_{i}\rangle \right), \end{array}$$

where $U_{CNOT}^{ij}$ denotes a controlled-NOT gate acting on qubit *i* (control) and *j* (target). Applying the quantum circuit U(x,θ) to the initial state |0〉 produces the final state $|\hat{x}\rangle$:

(22) $$\begin{array}{c} |\hat{x}\rangle =U\left(x,\theta \right)|0\rangle . \end{array}$$

This final state encodes both the classical input x and the variational parameters θ, and serves as the basis for subsequent measurements to obtain the model output. Then the output of the quantum model is obtained by measuring a Hermitian observable *O*, whose expectation value defines the model prediction [25]:

(23) $$\begin{array}{c} \langle O\rangle =\langle \hat{x}|O|\hat{x}\rangle , \end{array}$$

where *O* is a Hermitian operator representing the measured observable. We choose *O* to be a tensor product of Pauli-Z operators ${⨂}_{i=1}^{n}Z_{i}$, which allows efficient readout of the model output from the quantum state. In practice, 〈O〉 is estimated by repeated measurements of the observable *O* on multiple runs of the circuit.

### 2.5. Quantum-Encodable Bayesian PINNs Trained via Classical Ensemble Kalman Inversion

Gradient-based optimization of QNNs often encounters the barren plateau phenomenon, where the variance of gradients decays exponentially with the number of qubits or the depth of the circuit. As a result, randomly initialized circuits often produce gradients that are effectively zero, causing extremely slow or even stalled learning, particularly in deep or noisy quantum circuits. As a gradient-free update method, EKI naturally circumvents these difficulties by reducing the need for explicit gradient computation, thus avoiding the gradient concentration problems associated with barren plateaus, which enhances the training stability and accelerates the overall optimization of the QNN.

Motivated by these advantages, we incorporate QNNs into the EKI–BPINNs framework as surrogate models to solve PDE inverse problems. In this framework, the QNN surrogate approximates the forward solution of the governing PDE. Its parameters and the unknown PDE parameters are treated as part of the ensemble, which evolves during the inversion process. The EKI update infers the unknown physical parameters by assimilating noisy observational data while maintaining ensemble diversity. Furthermore, to fully exploit the input data for the QNN, we first perform a classical preprocessing step that constructs the network inputs as learnable linear combinations of the raw PDE data. Specifically, the QNN input $H_{in}$ is defined as $H_{in}=H_{0}W+b$, where $H_{0}$ represents the raw data, including residual data, boundary-condition data and solution measurement, and *W* and *b* are learnable parameters, which serve as a classical linear layer to map the raw data into a vector whose dimension matches the number of qubits used in the quantum circuit.

Algorithm 2 summarizes the complete training and inversion procedure. In this study, all computational stages are implemented and executed on classical hardware, where the QNN is simulated using the PennyLane v0.43 [26] quantum circuit simulator. In the current framework, the inversion algorithm, ensemble perturbation, and residual-based updates are entirely classical, while the quantum role is confined to the QNN-based surrogate representation and its circuit evaluation.

The classical–quantum interaction is organized as an iterative parameter-update scheme. In each step, the BPINNs input is mapped to a quantum state using angle encoding, and the parameterized variational layers transform the state according to the current QNN weights. The circuit is evaluated repeatedly with several measurement shots to estimate expectation values, which serve as the circuit output. These outputs are passed to the classical EKI routine, where the residual between the observations and the quantum surrogate predictions is used to generate the next ensemble update.

Within B-PINNs, the parameters $\xi =\left\{\theta ,\lambda \right\}$ are considered, with the forward operator G defining the model mapping. The quantity $y_{i}^{j}$ represents the result of evaluating the forward model $G(\xi_{i}^{j})$ in one step, which is defined as

(24) $$\begin{array}{c} y_{i}^{j}=G\left(\xi_{i}^{j}\right)=\left[\begin{array}{c} u(x;\theta_{i}^{j})\\ N_{x}\left(u\left(x;\theta_{i}^{j}\right);\lambda_{i}^{j}\right)\\ B_{x}\left(u\left(x;\theta_{i}^{j}\right);\lambda_{i}^{j}\right) \end{array}\right]. \end{array}$$

**Algorithm 2** Quantum-Encodable Bayesian PINNs trained via Classical Ensemble Kalman Inversion (QEKI)**Require:** Observations $y_{\mathrm{obs}}$, initial *J* ensemble states ${\left\{\xi_{0}^{j}\right\}}_{j=1}^{J}$, observation covariance *R*, parameter covariance *Q*, training data points x, iteration index i=0.    **while** not converge **do**        **for** j=1,2,…,J **do**            Sample $\epsilon_{i}^{j}$ from N(0,Q)            Update each ensemble state $\xi_{i}^{j}\leftarrow \xi_{i}^{j}+\epsilon_{i}^{j}$            $\left(\theta_{i}^{j},\lambda_{i}^{j}\right)\leftarrow \xi_{i}^{j}$            Apply quantum circuit $|\hat{x}\rangle \leftarrow U\left(x,\theta \right)|0\rangle$            Evaluate the expectation value ${\langle O\rangle }_{i}^{j}\leftarrow \langle \hat{x}\left|O\right|\hat{x}\rangle$            $y_{i}^{j}\leftarrow N_{x}\left({\langle O\rangle }_{i}^{j};\lambda_{i}^{j}\right)$        **end for**        $C_{i}^{yy}\leftarrow \frac{1}{J-1}\sum_{j=1}^{J}\left(y_{i}^{j}-{\bar{y}}_{i}\right){\left(y_{i}^{j}-{\bar{y}}_{i}\right)}^{T}$        $C_{i}^{\xi y}\leftarrow \frac{1}{J-1}\sum_{j=1}^{J}\left(\xi_{i}^{j}-{\bar{\xi }}_{i}\right){\left(y_{i}^{j}-{\bar{y}}_{i}\right)}^{T}$        **for** j=1,2,…,J **do**            Sample $\eta_{i}^{j}$ from N(0,R)            $\xi_{i+1}^{j}\leftarrow \xi_{i}^{j}+C_{i}^{\xi y}{\left(C_{i}^{yy}+R\right)}^{-1}\left(y_{\mathrm{obs}}+\eta_{i}^{j}-y_{i}^{j}\right)$        **end for**    **end while**    
$\hat{I}\leftarrow i+1$    **Return:** $\xi_{\hat{I}}^{1},\ldots ,\xi_{\hat{I}}^{J}$

Correspondingly, the observations defined in (2) can be formulated as follows:

(25) $$\begin{array}{c} y_{\mathrm{obs}}={\left[\begin{array}{c} {\left\{u^{i}\right\}}_{i=1}^{N_{u}}\;{\left\{f^{i}\right\}}_{i=1}^{N_{f}}\;{\left\{b^{i}\right\}}_{i=1}^{N_{b}} \end{array}\right]}^{T}. \end{array}$$

The physics-based residuals are evaluated via PennyLane’s interface, which maps the quantum circuit to a classically differentiable node. This setup enables the use of classical automatic differentiation while maintaining compatibility with native quantum gradient methods, such as the parameter-shift rule, for future execution on quantum processing units.

The choice of covariance matrices *Q* and *R* can significantly affect the performance of the algorithm. Previous studies have explored automatic estimation techniques for these matrices. In this work, following the strategy proposed in Ref. [6], we set *R* to be the covariance matrix as defined in (5):

(26) $$R=\left[\begin{array}{ccc} \sigma_{u}^{2}I_{N_{u}} & 0 & 0\\ 0 & \sigma_{f}^{2}I_{N_{f}} & 0\\ 0 & 0 & \sigma_{b}^{2}I_{N_{b}} \end{array}\right]$$

An appropriate choice of *Q* is essential to preserve ensemble spread, so we set *Q* to be

(27) $$Q=\left[\begin{array}{cc} \sigma_{\theta }^{2}I_{N_{\theta }} & 0\\ 0 & \sigma_{\lambda }^{2}I_{N_{\lambda }} \end{array}\right]$$

where $\sigma_{\theta }$ and $\sigma_{\lambda }$ represent the standard deviations of the artificial process noise introduced to preserve ensemble spread for the QNN parameters θ and the physical parameters λ, respectively.

To determine when QEKI iterations should terminate, we employ a discrepancy-based stopping rule inspired by classical iterative regularization methods. The basic idea is to monitor how well the prediction of the current ensemble-averaged model matches the observed data, measured by a weighted residual norm as defined in Ref. [6]. Let

(28) $$\begin{array}{c} D_{i}=∥R^{-1/2}\left(y_{\mathrm{obs}}-\frac{1}{J}\sum_{j=1}^{J}y_{i}^{j}\right)∥, \end{array}$$

denote the discrepancy at iteration *i*, where $y_{i}^{j}$ is the predicted observation produced by the *j*-th ensemble member at iteration *i*. Specifically, we define a sliding window of length *W* and terminate the iteration once the discrepancy no longer exhibits sufficient improvement, i.e.,

(29) $$\begin{array}{c} \max_{j\in \{i-W,\ldots ,i\}}\frac{\left|D_{j}-D_{i}\right|}{D_{i}}<\tau . \end{array}$$

Complexity Analysis. The computational cost of QEKI is lower than that of HMC, as it performs direct ensemble updates. The per-iteration computational complexity of QEKI is given by:

(30) $$\begin{array}{c} O\left(N_{y}^{3}+JN_{y}N_{\xi }+JN_{y}^{2}\right). \end{array}$$

This complexity can be decomposed into the following components:

(1) $O(JN_{y}^{2})$—Cost of constructing the observation covariance matrix $C_{i}^{yy}$
(2) $O(JN_{y}N_{\xi })$—Cost of constructing the observation covariance matrix $C_{i}^{\xi y}$
(3) $O(N_{y}^{3}+JN_{y}N_{\xi }+JN_{y}^{2})$—Cost of updating all ensemble members via the Kalman gain, $C_{i}^{\xi y}{\left(C_{i}^{yy}+R\right)}^{-1}\left(y_{\mathrm{obs}}+\eta_{i}^{j}-y_{i}^{j}\right)$.

In conclusion, the computational analysis indicates that the primary bottleneck of QEKI lies in the observation dimension $N_{y}$, specifically due to the $O(N_{y}^{3})$ cost associated with the matrix inversion in the Kalman gain. Conversely, the algorithm exhibits linear scalability with respect to both the high-dimensional parameter space $N_{\xi }$ and the ensemble size *J*. This structural characteristic allows for rapid ensemble updates without prohibitive computational costs.

This completes the description of the proposed QEKI framework. The next section presents numerical experiments that demonstrate its performance on representative PDE inverse problems.

## 3. Experiments

In this section, we present the experimental setup and numerical studies to evaluate the performance of the proposed QEKI framework. To ensure a fair and systematic comparison, we use the classical HMC-based B-PINNs as a baseline. This allows us to evaluate how well the quantum-encodable architecture can reproduce the posterior distribution under a well-understood classical inversion procedure. We note that due to current quantum-training bottlenecks and hardware limitations, QEKI does not achieve the same training speed as classical EKI-trained DNN surrogates. Nevertheless, a key advantage of the proposed approach is that it can maintain comparable posterior accuracy while using fewer trainable parameters, demonstrating the representational efficiency of the quantum-encodable architecture. All computations are executed on the open-source PennyLane v0.43 [26] and JAX 0.8.0 [27] platforms on a single GPU (NVIDIA GeForce RTX 4090 with 24 GB of memory).

In the HMC case, we adopt the variant of HMC described in [28]. The surrogate model is a neural network with two hidden layers, each containing 50 neurons and equipped with the activation function tanh. The number of neural network parameters is listed for each experiment. For the HMC implementation, the leapfrog integration step is set to I=50, the initial time step is δt=0.1, the burn-in period consists of 1000 steps, and a total of 2000 posterior samples are collected.

In the EKI case, we employ a QNN architecture, as described in Ref. [14], consisting of four qubits and six entangling layers, while in Experiment 3, we use a circuit with six qubits and eight entangling layers to accommodate the increased model complexity introduced by the nonlinear advection term in the Burgers equation. The size of the ensemble is set to J=200, and the initial ensemble states ${\left\{\xi_{0}^{j}\right\}}_{j=1}^{J}$ are drawn from the prior distribution, the standard deviations of artificial dynamics of the parameters are chosen to be $\sigma_{\lambda }=0.01$ and $\sigma_{\theta }=0.01$. For the stopping rule, we choose the size of the sliding window to be W=25 and the tolerance τ=0.05.

Synthetic observation data are generated by first solving the target PDEs and then adding Gaussian noise with two different levels ($\sigma_{u}=\sigma_{f}=\sigma_{b}$ = 0.1 and 0.01) to test robustness against observation uncertainty. For the 1D problems, both the solution measurements and the PDE residual points are placed on uniformly spaced grids. For the 2D examples, the solution measurements are randomly sampled in the physical domain, while the residual points inside the domain are generated using Latin hypercube sampling, and the boundary points are uniformly sampled along the boundary. The priors are all standard Gaussian distributions with zero mean.

To assess the accuracy of the solution and the estimation of the physical parameter, we compute the following error:

(31) $$e_{u}=\frac{∥u-\bar{u}{∥}_{L_{2}}}{{∥u∥}_{L_{2}}},\;e_{\lambda }=\frac{∥\lambda -\bar{\lambda }{∥}_{L_{1}}}{{∥\lambda ∥}_{L_{1}}},$$

where u and λ are the reference solution and reference physical parameter, $\bar{u}$ and $\bar{\lambda }$ are the sample means of the approximate solution and approximate physical parameter. In all experiments considered in this work, we restrict λ to a scalar parameter. To evaluate computational performance, we compare the mean walltime of QEKI and HMC over 10 independent trials. We further compare the number of trainable parameters, with the numbers in parentheses indicating the total number of parameters in the linear classical layers at the input of the QNN.

### 3.1. One-Dimensional Nonlinear Poisson Equation

We first consider the 1D nonlinear Poisson problem:

(32) $$\lambda u_{xx}+k\tanh\left(u\right)=f,\;x\in \left[-0.7,0.7\right].$$

In this experiment, we set λ=0.01, and set k=0.7 as the unknown physical parameter. The forward problem admits the analytical solution $u\left(x\right)=sin^{3}\left(x\right)$, from which the source term and the boundary conditions are exactly derived. The inverse task is to recover the parameter *k* and the solution u(x), together with their associated uncertainty estimates. To enforce the PDE constraints, we employ 8 interior points, 2 boundary points, and 32 collocation points.

As shown in Table 1, both QEKI and HMC achieve an accurate recovery of the physical parameter *k*, with the true value k=0.7 consistently lying within one standard deviation band of their posterior mean estimates in both noise settings. This indicates that both Bayesian frameworks can provide statistically reliable uncertainty quantification for the inverse problem.

**Figure 3:**
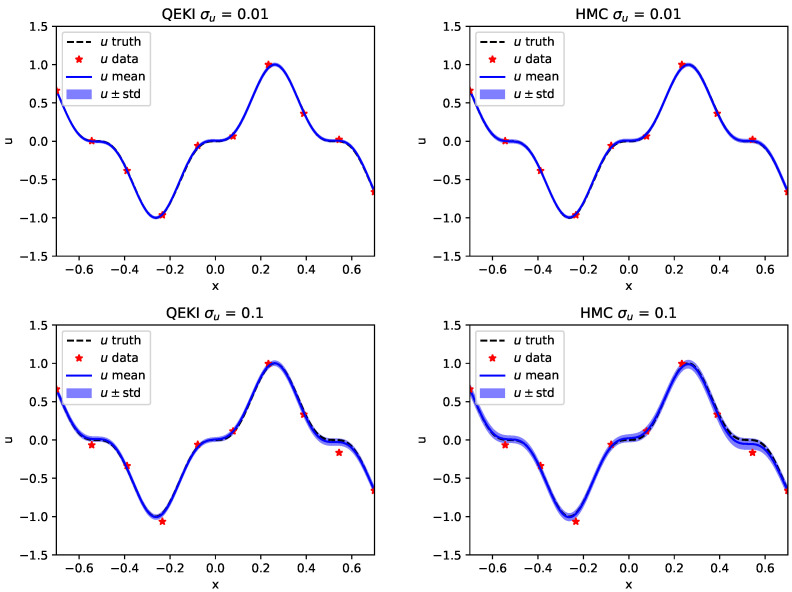
Section 3.1: Reference solution, observation samples, sample mean and standard deviation by QEKI and HMC for $\sigma_{u}$ = 0.01, 0.1 noise levels.

Figure 3 further compares the sample mean and standard deviation of the reconstructed surrogate solutions; the QEKI results show a consistently smaller posterior variance.

Table 2 further reports the relative errors in the estimated means of *u* and *k*, together with the computational walltime and the number of trainable parameters for each method. Notably, QEKI achieves comparable or better accuracy while requiring substantially fewer trainable parameters and reduced computational cost, highlighting its potential as a lightweight yet effective quantum-assisted inversion framework.

### 3.2. Two-Dimensional Nonlinear Diffusion–Reaction Equation

Here, we consider the following 2D nonlinear PDE:

(33) $$\begin{array}{cc} \lambda \Delta u+ku^{2} & =f,\;\left(x,y\right)\in {\left[-1,1\right]}^{2},\\ u(x,-1) & =u(x,1)=0,\\ u(-1,y) & =u(1,y)=0. \end{array}$$

**Figure 4:**
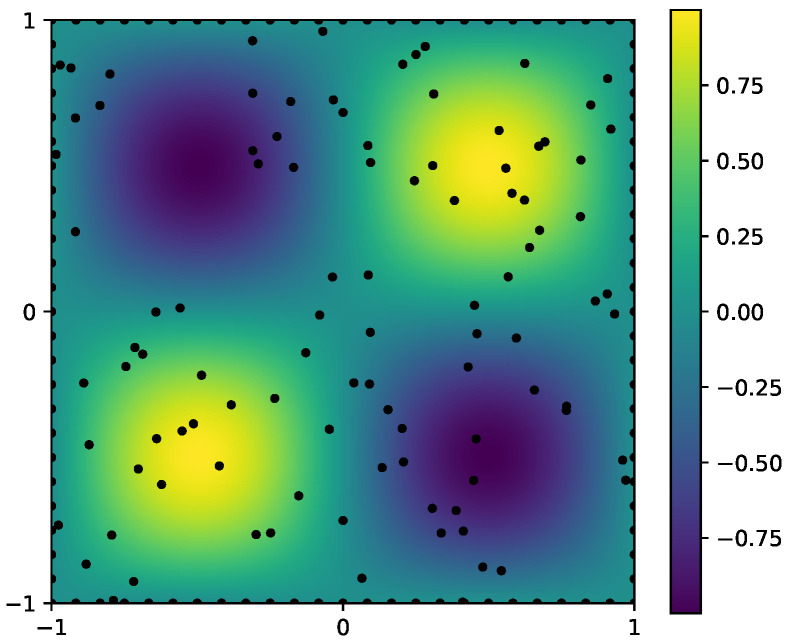
Section 3.2: Observation points of the forward solution and the corresponding boundary measurements (represented by black dots).

In this experiment, we set λ=0.01 and set k=1 as the unknown physical parameter to be inferred. The forward problem admits the analytical solution u(x)=sin(πx)sin(πy), from which the source term and the boundary conditions are analytically derived. The inverse problem aims to recover both the parameter *k* and the solution u(x) with uncertainty estimates. To enforce the PDE constraints, we employ 100 interior points, 100 boundary points, and 100 collocation points. The true solution and the observation points are illustrated in Figure 4.

Table 3 shows that both QEKI and HMC successfully recover the physical parameter *k*, with the true value k=1 consistently lying within the two standard deviation bands of their posterior mean estimates in both noise settings. This indicates that both Bayesian frameworks provide reliable uncertainty quantification for the inverse problem.

**Figure 5:**
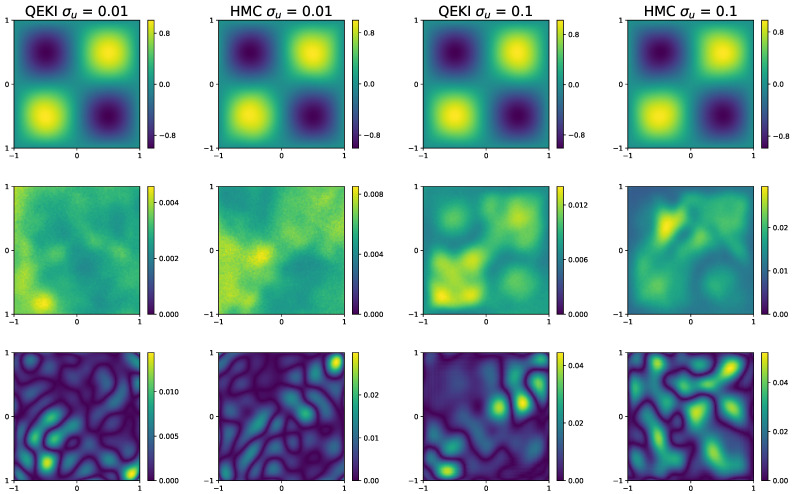
Section 3.2: Comparison of QEKI and HMC under different noise levels $\sigma_{u}=0.01,0.1$. The prediction values obtained by both methods (**top row**), the corresponding standard deviations (**middle row**), the absolute error between the ground-truth solution (**bottom row**).

Figure 5 compares the sample mean and standard deviation of the surrogate solutions obtained by the two approaches. The QEKI mean closely matches the reference solution, demonstrating its effectiveness in reconstructing the forward solution, while HMC similarly captures the overall trend but exhibits slightly larger posterior variance.

Table 4 shows the relative error of the estimated means for both *u* and *k*, along with the computational walltime and the number of trainable parameters for each method. Notably, QEKI achieves comparable or slightly better accuracy while requiring fewer trainable parameters and reduced computational cost, highlighting its potential as a computationally efficient quantum-assisted inversion framework. These results collectively demonstrate that QEKI can provide accurate and stable posterior estimates for both the physical parameter and the surrogate solution, even under different noise levels.

### 3.3. Burgers Equation

**Figure 6:**
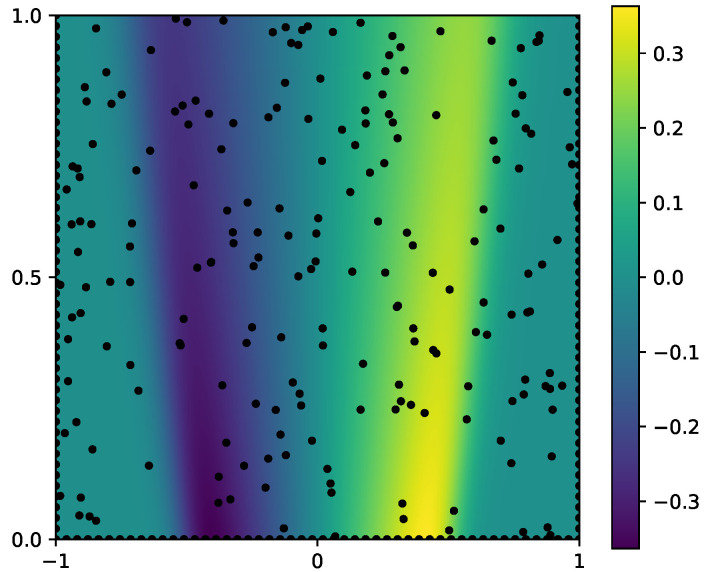
Section 3.3: Observation points of the forward solution and the corresponding initial and boundary measurements (represented by black dots).

Here, we consider the following Burgers equation as presented in Ref. [15]:

(34) $$u_{t}+uu_{x}=\nu u_{xx},$$

with a viscosity of ν=0.01,which we set to be the unknown physical parameter, in a computational domain of (x,t)=[−1,1]×[0,1]. The exact solution under the Dirichlet boundary condition is given by

(35) $$u\left(x,t\right)=\frac{\frac{x}{t+1}}{1+\sqrt{\frac{t+1}{t_{0}}}e^{\frac{x^{2}}{4\nu (t+1)}}},$$

with $t_{0}=\exp\left(\frac{1}{8\mu }\right)$. The initial and boundary conditions can be derived exactly. To enforce the PDE constraints, we employ 50 initial points, 100 boundary points, 200 collocation points, and 200 interior points. The true solution and the observation points are illustrated in Figure 6.

Table 5 shows that both QEKI and HMC successfully recover the physical parameter ν, with the true value ν=0.01 consistently lying within the one standard deviation interval of the posterior mean under both noise levels. This indicates that both Bayesian inversion frameworks remain effective even in the presence of nonlinearity introduced by the Burgers equation.

**Figure 7:**
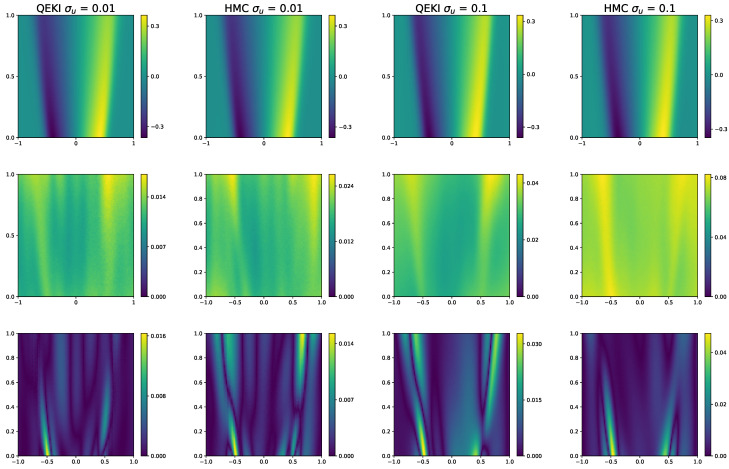
Section 3.3: Comparison of QEKI and HMC under different noise levels $\sigma_{u}=0.01,0.1$. The prediction values obtained by both methods (**top row**), the corresponding standard deviations (**middle row**), the absolute error between the ground-truth solution (**bottom row**).

Figure 7 further compares the surrogate predictions obtained from the two methods. The QEKI produces a mean solution that closely matches the reference, while the HMC captures the overall trend.

In addition, Table 6 summarizes the relative errors of the posterior mean estimates for both *u* and ν, together with the computational wall-time and the number of trainable parameters for each method. Notably, although the QNN architecture is enlarged for this nonlinear problem, the computational speed of QEKI experiences a slight decrease due to quantum hardware limitations, yet it still requires fewer trainable parameters compared to HMC. These results indicate that QEKI can reliably provide accurate posterior estimates for both the physical parameter and the surrogate solution, even in more challenging nonlinear scenarios, while maintaining overall computational efficiency.

### 3.4. Capacity Analysis

The focus of this experiment is to assess representational efficiency under a similar number of trainable parameters for the classical DNN-based method in comparison with the proposed QEKI architecture. In particular, we first examine whether the DNN model remains trainable with which architectures are constrained to a similar parameter budget as QEKI. In practice, we observe that the DNN-based method exhibits insufficient expressive power under this equal-capacity constraint and fails to achieve stable convergence, resulting in unreliable posterior estimates. In contrast, the QEKI model remains trainable and produces stable posterior inference under the same parameter budget. For this reason, additional DNN-based models with increased parameter counts are included to evaluate how much model capacity is required for classical architectures to recover comparable posterior accuracy and training behavior.

Specifically, we consider neural network architectures of three different sizes, corresponding to two-hidden-layer fully connected networks with 10, 30, and 50 neurons per hidden layer, resulting in 152, 1052, and 2752 trainable parameters, respectively. For each network configuration, both EKI and HMC are employed as inference engines. All experiments are conducted on the Burgers equation, as in Section 3.3, with the observation noise level fixed to 0.01. In all cases, the associated hyperparameters are selected following standard practice and kept consistent across models to ensure a fair comparison.

**Figure 8:**
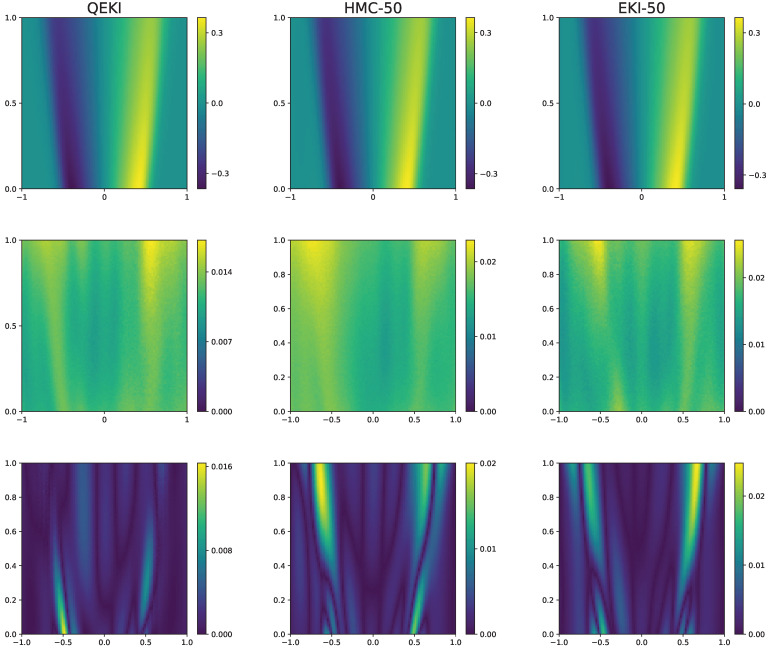
Comparison of QEKI and HMC, EKI with 50-neuron-DNN under noise levels $\sigma_{u}=0.01$. The prediction values obtained by these methods (**top row**), the corresponding standard deviations (**middle row**), the absolute error between the ground-truth solution (**bottom row**).

As shown in Table 7, we observe that networks with 10 and 30 neurons per layer fail to achieve stable convergence, while the network with 50 neurons per layer successfully converges and produces posterior estimates comparable to those obtained with QEKI, as shown in Figure 8. These results indicate that the QEKI architecture can maintain meaningful posterior inference under a much more restrictive parameter budget, highlighting the representational efficiency of QEKI.

## 4. Conclusions

This work introduced QEKI, a hybrid inversion framework that combines Ensemble Kalman Inversion (EKI) with a Quantum Neural Network (QNN) to solve PDE-based inverse problems. Experiments demonstrate that QEKI can achieve the same level of accuracy as classical EKI-based approaches while utilizing significantly fewer trainable parameters. To address current difficulties in training QNNs with gradients, EKI, a gradient-free optimization strategy for QNN parameter updates, demonstrates that reliable inversion can still be achieved without back-propagated quantum gradients.

The study shows promise, but there are still constraints that need to be addressed.

(1) The feasible circuit size is capped by the number of qubits and circuit depth. These two factors set the practical ceiling for what we can simulate today rather than the learning method itself. As the circuit grows, the simulation burden quickly outweighs the benefit of testing larger models.
(2) High-dimensional PDE inverse problems cannot yet be handled directly, since current QNN encoding capacity is limited by available qubits.
(3) The EKI update process can be unstable, and we observed that QEKI inherits this issue, leading to fluctuating convergence behavior in some cases.

Looking ahead, progress can be made even before quantum hardware scales up. A promising direction is to explore more efficient quantum network designs—for example, circuit ansatzes that are smaller, better structured, and tailored to PDE solution patterns. By improving how information and parameters are arranged in the circuit, we hope to expand simulation capacity without relying solely on adding qubits or depth. Second, strengthening the stability of the EKI update, for example, by adaptive ensemble re-scaling, improved noise modeling, or regularized Kalman updates, could lead to more predictable convergence. Finally, to evaluate high-dimensional inverse tasks under qubit constraints, dimensionality reduction techniques, such as KL expansion, VAE, or other latent-space parameterizations, can be incorporated, enabling QEKI to test surrogate inversion of high-dimensional parameters in a lower-dimensional space.

In summary, QEKI demonstrates that quantum models can achieve classical inversion accuracy with fewer parameters, and that QNNs can be optimized without requiring explicit quantum gradients. Future work should focus on improving model scalability, algorithm stability, and testing high-dimensional problems through the use of reduced representations.

## Figures and Tables

**Table 1:** Section 3.1: Sample mean and standard deviation of parameter *k* for QEKI and HMC for $\sigma_{u}$ = 0.01, 0.1 noise levels. The true value of *k* is 0.7.

		*k* (Mean ± Std)
$\sigma_{u}=0.01$	QEKI	0.699±0.006
	HMC	0.702±0.007
$\sigma_{u}=0.1$	QEKI	0.689±0.011
	HMC	0.687±0.018

**Table 2:** Section 3.1: Relative errors $e_{u}$ of the forward solution *u* and $e_{k}$ of parameter *k* for the noise levels $\sigma_{u}$ = 0.01, 0.1, average walltime and trainable parameters.

		$e_{u}$	$e_{k}$	Walltime (s)	Trainable Parameters
$\sigma_{u}=0.01$	QEKI	1.23%	0.14%	13.01	85(+8)
	HMC	1.13%	0.15%	45.32	5252
$\sigma_{u}=0.1$	QEKI	8.03%	1.57%	13.11	85(+8)
	HMC	9.21%	1.85%	46.02	5252

**Table 3:** Section 3.2: Sample mean and standard deviation of the parameter *k* for QEKI and HMC for $\sigma_{u}$ = 0.01, 0.1 noise levels. The true value of *k* is 1.

		*k* (Mean ± Std)
$\sigma_{u}=0.01$	QEKI	0.992±0.008
	HMC	0.988±0.012
$\sigma_{u}=0.1$	QEKI	1.029±0.022
	HMC	0.964±0.035

**Table 4:** Section 3.2: Relative errors $e_{u}$ of the forward solution *u* and $e_{k}$ of the unknown parameter *k* for noise levels $\sigma_{u}$ = 0.01, 0.1, including average walltime and number of trainable parameters.

		$e_{u}$	$e_{k}$	Walltime (s)	Trainable Parameters
$\sigma_{u}=0.01$	QEKI	1.03%	0.88%	23.42	85(+12)
	HMC	1.21%	1.21%	51.22	5302
$\sigma_{u}=0.1$	QEKI	2.77%	3.41%	24.52	85(+12)
	HMC	2.82%	3.62%	52.03	5302

**Table 5:** Section 3.3: Sample mean and standard deviation of the parameter ν for QEKI and HMC for $\sigma_{u}$ = 0.01, 0.1 noise levels. The true value of ν is 0.01.

		$\nu \;(\times 10^{3})$ (Mean ± Std)
$\sigma_{u}=0.01$	QEKI	10.257±0.327
	HMC	9.719±0.502
$\sigma_{u}=0.1$	QEKI	10.614±0.823
	HMC	10.740±0.908

**Table 6:** Section 3.3: Relative errors $e_{u}$ of the forward solution *u* and $e_{\nu }$ of the unknown parameter ν for noise levels $\sigma_{u}$ = 0.01, 0.1, including average walltime and number of trainable parameters.

		$e_{u}$	$e_{\nu }$	Walltime (s)	Trainable Parameters
$\sigma_{u}=0.01$	QEKI	1.41%	2.57%	44.88	163(+18)
	HMC	1.58%	2.81%	56.33	5302
$\sigma_{u}=0.1$	QEKI	4.22%	6.41%	45.03	163(+18)
	HMC	4.72%	7.41%	56.09	5302

**Table 7:** Comparison of QEKI and EKI, HMC with classical DNNs of different sizes under noise level $\sigma_{u}$ = 0.01, together with convergence, relative errors $e_{u}$ and $e_{\nu }$ of the unknown parameter ν.

	Trainable Parameters	Converged	$e_{u}$	$e_{\nu }$
QEKI	163 (+18)	yes	1.41%	2.57%
HMC	152	no	-	-
	1052	no	-	-
	2752	yes	1.68%	2.88%
EKI	152	no	-	-
	1052	no	-	-
	2752	yes	1.72%	2.97%

## Data Availability

Data is contained within the article.
